# Beyond the initial impact: troponin patterns frequently reveal delayed cardiac injury in polytrauma patients

**DOI:** 10.1186/s13017-026-00672-4

**Published:** 2026-01-31

**Authors:** Larissa Sztulman, Victoria Pfeiffer, Miriam Saenger, Ruth Brenner, Lea Usov, Ingo Marzi, Birte Weber

**Affiliations:** 1 Department of Trauma Surgery and Orthopedics, Goethe University Frankfurt, University Hospital, 60590 Frankfurt, Germany; 2 Department of Cardiology, Goethe University Frankfurt, University Hospital, 60590 Frankfurt, Germany

## Abstract

**Background:**

Cardiac troponin serves as a biomarker for diagnosing myocardial contusion following blunt chest trauma and for differentiating between types of myocardial infarction. However, its interpretation in polytrauma remains challenging due to overlapping pathophysiological mechanisms. This study aims to improve troponin-based cardiac risk stratification to more accurately identify high-risk patients and enhance prognostic assessment.

**Methods:**

This prospectively performed study included polytraumatized patients (ISS ≥ 16) admitted to a German Level 1 trauma center between January 2024 and July 2025. For each patient, six blood samples collected over ten days were analyzed for Troponin T (TnT) and NT-proBNP; and two transthoracic echocardiograms (24 h and 48 h) and ECGs were evaluated by a cardiologist. Data were correlated with clinical records, trauma-dependent and -independent cardiac risk factors, including the cardiovascular risk score (SCORE2).

**Results:**

Seventy-seven patients were included (mean age 52 years; 73% male; mean ISS 29). TnT was elevated in 44% at admission and in 73% after 24 h. 13% of the patients were diagnosed with a cardiac contusion. TnT elevation was associated with age ≥ 40 years, higher SCORE2, thoracic injuries, ISS ≥ 25, preclinical arrhythmias, catecholamine therapy, and surgery at admission. Two distinct TnT patterns were found: Group 1 (44%)—elevation already at admission, mirrored the overall risk profile but showed more persistent elevation in patients ≥ 60 years, with very high SCORE2 or catecholamine therapy and was especially linked to sternal fractures. Group 2 (26%)—delayed TnT rise after 24 h, associated with thoracic trauma, ISS ≥ 25, surgery and catecholamine therapy. Complications, including new-onset arrhythmias and higher mortality, occurred in both groups.

**Conclusion:**

Cardiac involvement in polytrauma is multifactorial and often underrecognized. TnT elevation was associated with higher age, high SCORE2, severe injury, thoracic trauma, arrhythmias, and resuscitation, with a distinct subgroup showing delayed elevation after 24 h. This delayed phenotype is clinically relevant, as most of these patients had thoracic trauma and underwent early surgery, aligning with recommendations for perioperative screening for myocardial infarction. Our findings emphasize routine peri-traumatic and peri-operative troponin measurement and highlight the value of TTE and continuous ECG for detecting evolving cardiac dysfunction. Systematic follow-up is needed to assess long-term outcomes and refine cardiac risk stratification in this vulnerable population.

**Supplementary Information:**

The online version contains supplementary material available at 10.1186/s13017-026-00672-4.

## Introduction

Polytrauma is defined through an Injury Severity Score (ISS) ≥ 16. The Berlin Definition, more specifically, defines polytrauma as severe injuries (AIS ≥ 3) to at least two body regions accompanied by one or more critical physiological risk factors such as hypotension, low Glasgow Coma Scale, metabolic acidosis, coagulopathy, or advanced age. The resulting systemic inflammatory response (SIRS) can drive secondary organ failure [1, 2]. Especially thoracic trauma is highly relevant in the context of severe injury: data from the German Trauma Register DGU® (2021–2023) show that approximately 45% of polytrauma patients sustained a thoracic trauma [3]. Blunt cardiac injury has been reported in ~ 10% of general trauma cases and up to 70% following high-energy thoracic trauma[4, 5]. Beyond the direct cardiac injury from blunt or penetrating mechanisms, polytrauma represents a unique pathophysiological setting with an additional burden of secondary cardiac damage due to SIRS. Following excessive catecholamine release, endothelial and microvascular dysfunction, immune dysregulation, oxidative stress, and myocardial oxygen supply–demand imbalance (characteristic of type 2 myocardial infarction) may all contribute to indirect cardiac damage [6, 7]. Consequently, clinical manifestations range widely—from sudden cardiac death or the need for emergent surgical intervention, to arrhythmias, functional impairment, or entirely asymptomatic courses [4]. This heterogeneity substantially complicates clinical recognition and underscores the need for structured cardiac evaluation to identify high-risk patients. For this reason, the current S3 polytrauma guidelines emphasize the early detection of cardiac injury, particularly cardiac contusion [8]. Recommended diagnostics include electrocardiogram (ECG) monitoring and troponin (Tn) measurement as admission gold standard, combined with targeted trauma imaging [8, 9]. Numerous studies have demonstrated that elevated Tn levels in trauma are associated with poorer clinical outcomes, prolonged intensive care unit stays, and extended durations of mechanical ventilation [10, 11]. However, the diagnostic utility of Tn is limited by its lack of specificity, as elevations may arise from both cardiac and non-cardiac causes [12]. In cardiology, Tn has long been considered the gold standard for diagnosing myocardial infarction (MI) and non-ischemic myocardial injury [13]. With the advent of high-sensitivity assays, interpretation has been refined, and the “Fourth Universal Definition of Myocardial Infarction” was introduced to differentiate Tn elevations into distinct etiological categories [14, 15]. However, the frequent use of the type 2 MI category as a “catch-all” for non-thrombotic injury often results in diagnostic uncertainty, and trauma as a special etiology for Tn dynamics is not included in that definition [13].

Tn elevation in polytrauma may arise via multiple mechanisms, leading to a more heterogeneous pattern than in studies limited to blunt chest trauma. Known mechanisms—such as type 1 and type 2 MI (including tachyarrhythmia, acute heart failure, and extracardiac stressors such as anemia, hypoxemia, or catecholamine exposure)—overlap with non-ischemic myocardial injury caused by direct trauma, systemic inflammation, and immune dysregulation in polytrauma patients [6]. Furthermore, in an aging population with rising cardiovascular risk burden, attributing Tn dynamics to a specific etiology becomes increasingly difficult, complicating the development of targeted diagnostic or therapeutic strategies. Accordingly, a clear etiology of Tn elevation in the polytrauma collective remains elusive, and interpretation as a gold standard requires considerable expertise [12, 16]. Additional complexity arises from the risk of peri-operative myocardial infarction/injury (PMI), which is also defined by perioperative Tn dynamics. A cardiovascular discourse is currently underway regarding the lack of a definition for PMI, despite evidence that surgical patients with PMI have a substantially increased risk of cardiovascular events within one year [17–19]. PMI may be triggered by surgical trauma, anesthesia, sympathetic activation, hypercoagulability, and systemic inflammation [17–19]—which are especially appearing in polytrauma [6]. While this is recognized in non-cardiac surgery, the special implications for polytrauma patients remain unexplored, even though they carry similar or greater risks for cardiovascular complications [17, 20]. Therefore, the aim of this study is to investigate Tn dynamics in polytrauma and their association with trauma-dependent and independent risk factors, as well as overall cardiac complications. A further objective is to evaluate whether Tn alone is a reliable marker of cardiac contusion or dysfunction in polytrauma, or whether confounding factors necessitate repeated measurements and additional diagnostic tools to identify patients at risk for adverse cardiac outcomes.

## Materials and methods

### Study design

This study followed a prospective, non-randomized design and was conducted at a German Level 1 trauma center between January 2024 and July 2025. Polytraumatized patients with an ISS ≥ 16 and of legal age (*n* = 77) were included immediately after entering the emergency department. Entering the intensive care unit, transthoracic echocardiography (TTE) was performed at two time points (after 24 h and 48 h), by an experienced cardiologist. Blood samples were withdrawn during routine diagnostics at 6 timepoints: at the Emergency room, after 24 h, 48 h, 72 h, at the 5th and 10th day of hospitalization. Digital patient records served as the source for clinical and demographic variables, which were subsequently evaluated against routine laboratory findings and published reference standards.

### Cardiac damage markers

Serum Troponin T and NT-proBNP concentrations were measured via highly sensitive electrochemiluminescence immunoassays (ECLIA, Roche, Rotkreuz, Switzerland). At this German Trauma Center, high-sensitivity TnT is the standard biomarker used for the evaluation of cardiac injury in thoracic trauma and for myocardial infarction; therefore, TnT was consistently available and selected for this study.

### Transthoracic Echocardiography and Electrocardiogram Evaluation

At arrival to the surgical intensive care unit, TTE was performed twice by an experienced cardiologist—within the first 24 h and again 24–48 h later. The assessment included an evaluation of left and right ventricular function, chamber dimensions, wall thickness, valvular function, and detection of pericardial or pleural effusion [21]. Examinations were conducted in cooperation with the Department of Cardiology at the University of Frankfurt, following the standards of the German Centre for Cardiovascular Research (DZHK), using the same GE HealthCare devices (Venue Go R2 and Vivid iq). ECGs were evaluated retrospectively by reviewing patient records, comparing admission ECGs with all subsequent recordings.

#### SCORE 2

The preclinical cardiovascular risk profile was assessed using SCORE2, a tool recommended by the European Society of Cardiology, based on age, blood pressure, smoking status, HDL and LDL cholesterol, and sex. This score estimates the 10-year risk of major cardiovascular events and stratifies patients into low-, high-, or very-high-risk categories [22].

### Subgroup Analysis

Based on observed Tn dynamic patterns and current discussions regarding the definition of peri-operative myocardial infarction [17, 20], two subgroups were identified and analyzed for trauma-dependent and -independent risk factors: patients with Tn elevation at admission and those with delayed elevation after 24 h. An elevation was defined by an increase above the 99th percentile, hence ≥ 14 pg/ml.

### Adverse Biomarker Analysis: Hemoglobin, Interleukin- 6 and Creatinine

In parallel with serial measurements of the cardiac biomarkers, additional clinically relevant biomarkers, which may also play a role in TnT elevation, were monitored over the 10-day observation period, including interleukin-6, renal function parameters, lactate, and hemoglobin (Hb). Subsequently, associations between cardiac biomarkers and these parameters were analyzed. Since anemia may lead to type 2 MI, the Hb level measured at the time point of cardiac biomarker sampling were analyzed separately and additional subgroup analysis were performed.

## Ethical approval

Ethical approval was granted by the local ethics committee of the University of Frankfurt (approval ID 89/19). Written informed consent was acquired from all enrolled patients or their legal guardian.

### Statistical analysis

Statistical analyses were conducted by excel and with Graph Pad-Prism 10 (Dotmatic, San Diego, CA, USA). Before subsequent analyses, data were tested for normal distribution using D’Agostino-Pearson and Shapiro Wilk tests. Multivariate analysis were then performed using Kruskal–Wallis multiple comparison test with Dunn’s correction. For the analysis of two isolated groups, the Mann–Whitney U test was performed. In case of significance within a group or within the same timepoint, results were marked. Correlation analysis was carried out with the spearman rank correlation. Analysis with a p-value ≤ 0.05 were considered significant. Data are presented as median and interquartile range.

## Results

### Patient characterization

The present study included 77 patients. In accordance with the German Trauma Registry most of the patients were male (56 vs. 21 female). The mean ISS was 29 ± 14 standard deviation (SD). The mean age was 52 ± 18 SD years. The most common *pre-existing conditions* (Additional file 1) were arterial hypertension (35.1%) and psychiatric diseases (33.8%). *Thoracic injury* was present in 51 (66.2%) patients: 40 (51.9%) without sternal fracture, 8 (10.4%) with an associated sternal fracture, and 3 (3.9%) as isolated sternal fractures. According to the guidelines, 13% of the patients were diagnosed with a cardiac contusion. The leading *trauma mechanisms* (Additional file 2) were falls from > 3 m (27.3%), followed by falls from < 3 m (14.3%), motorbike accidents (13.0%), and crush injuries (11.7%).

### Complications & Mortality

Of 77 patients, 10 patients died (13%). The most common reason for death was a major traumatic brain injury. 8 of the non-survivors were 60 years or older. 7 had a thoracic injury (2 including a sternal fracture). 4 of 10 had suffered an acute coronary syndrome before, of which 3 had to be reanimated. The most common trauma mechanism was a fall (9 of 10). 8 out of 10 patients had a high or very-high cardio-vascular risk profile based on the “SCORE 2”. 7 showed a TnT elevation at admission and 3 developed a delayed TnT elevation after 24h. In the overall population the most frequent in-hospital complications were infections (41.6%), shock (36.4%), and acute respiratory distress syndrome (29.9%). New onset atrial fibrillation occurred in 6,5%.

## Overall analysis of troponin dynamics

**Fig. 1:**
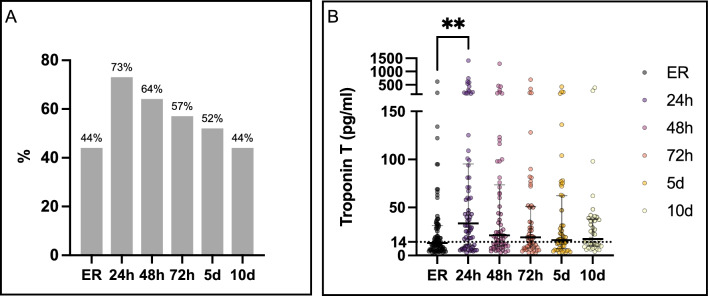
Frequency of TnT Elevation (**A**) and median TnT concentrations with interquartile ranges (**B**). The bar chart shows the percentage of patients with elevated TnT at admission (ER), 24 h, 48 h, 72 h, 5 d, and 10 d. Multigroup comparisons were performed using the Kruskal–Wallis test with Dunn’s post hoc correction. The dotted line indicates the 99th percentile threshold (14 pg/mL). ** p < 0.01; *n* = 77

At admission approximately 44% of the patients showed a TnT elevation, after 24 h a peak with 73% appeared with a slow decrease within the following days. On day 10 still 44% showed a TnT elevation above the 99th percentile (Fig. 1a). Both median (Fig. 1b) and mean TnT concentrations increased within the first 24 h and declined thereafter. Median values increased from 13.0 to 33.5 pg/mL, while mean concentrations peaked at 112.3 ± 220.5 pg/mL. Substantial interindividual variability persisted across all time points (coefficient of variation > 150%), with consistently right-skewed distributions, hence higher mean, than median values.

### Trauma independent factors

**Fig. 2:**
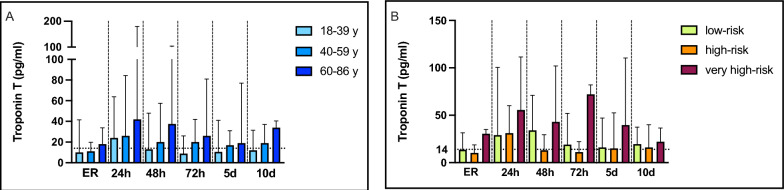
Trauma independent risk factors age (**A**) and SCORE 2 (**B**) for Troponin T elevation ER = emergency room, h = hours, d = day. Bars represent median values with interquartile ranges. Multivariate analysis was performed using the Kruskal–Wallis test with Dunn’s correction for multiple comparisons. The dotted horizontal line represents the 99th percentile threshold of TnT values (14 pg/ml). *p < 0.05

Potential trauma-independent risk factors for Tn elevation subgroup analyses were performed. Age (Fig. 2a): Since Tn elevation is known to be more prevalent in the elderly due to comorbidities, the influence of age was analyzed. Based on the distribution of patients and aligned with the the cardiovascular risk profiles from the Framingham Heart Study [23], three age groups were defined: 18–39 years (*n* = 21), 40–59 years (*n* = 28), and 60–86 years (*n* = 28). All three groups showed median Tn levels above the 99th percentile after 24 h, prolonged Tn elevation was observed in the elderly, with an additional peak on day 10. Cardiovascular risk—SCORE2 (Fig. 2B): To further specify individual cardiovascular risk profiles, patients were stratified according to the SCORE2 algorithm into low-risk (*n* = 34), high-risk (*n* = 32), and very high-risk (*n* = 10) groups. In the very high-risk group, a difference was detected compared with the other groups yet not receiving statistical significance, potentially based on a smaller group size. Interestingly, 80% in this group presented with elevated Tn at admission, and a secondary rise was observed after 72 h. Gender: Given the known sex-specific manifestations of acute MI, gender-specific analyses were conducted. Although most patients were male (*n* = 56 vs. *n* = 21 females), this distribution reflects typical trauma demographics. Both sexes demonstrated Tn elevations after 24 h, with statistical significance in the male group.

### Trauma dependent risk-factors

**Fig. 3:**
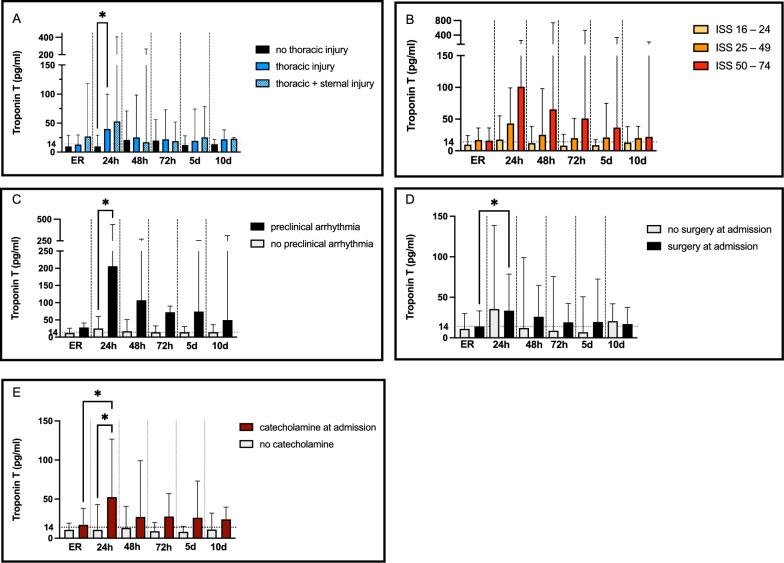
Trauma dependent risk factors: thoracic injury (**A**), ISS (**B**), arrhythmia (**C**), surgery (**D**) and catecholamine therapy (**E**). ER = emergency room, h = hours, d = day. Bars represent median values with interquartile ranges. Multivariate analysis was performed using the Kruskal–Wallis test with Dunn’s correction for multiple comparisons. The dotted horizontal line represents the 99th percentile threshold of TnT values (14 pg/ml). *p < 0.05

To further evaluate trauma-dependent determinants of Tn elevation, further analyses were performed (Fig. 3, A-E). Thoracic injury (Fig. 3a): Given the known association between thoracic trauma and myocardial injury, patients were stratified into three groups: no thoracic or sternal injury (*n* = 26), thoracic injury without sternal involvement (*n* = 40), and combined thoracic and sternal injury (*n* = 8). Three patients presented with isolated sternal fractures. A significant difference in Tn values was observed after 24 h in case of a thoracic injury. In patients with thoracic injury without sternal fracture, a significant TnT elevation was observed after 24 h, while those with sternal fractures most often presented with elevated values already at admission. Injury Severity Score (Fig. 3, B): The ISS was used to stratify injury burden into three categories: ISS 16–24 (*n* = 33), ISS 25–49 (*n* = 37), and ISS 50–75 (*n* = 7). Severe injury (ISS ≥ 25) was associated with higher and more sustained Tn elevations compared with lower ISS categories. Arrhythmia and resuscitation (Fig. 3**, C):** Eleven patients (14%) presented with arrhythmias at admission (including four with asystole), and nine required resuscitation preclinically or on admission. Both conditions were associated with markedly elevated Tn levels throughout the 10-day period. Resuscitation was required in one of seven patients with ISS ≥ 50 (14%) and in five of 37 with ISS 25–49 (14%). Surgery (Fig. 3D) and catecholamine therapy (Fig. 3E) at admission: Overall, 46 patients (60%) underwent surgery and 43 (56%) received catecholamine therapy at admission. A significant association between surgery at admission and elevated TnT levels was observed after 24 h. On average, TnT values in both groups remained above the 99th percentile throughout the 10-day observation period. *Hemoglobin:* Mean Hb levels declined significantly over time (ER: 12.30 ± 2.41 g/dL, 24 h: 10.78 ± 2.03 g/dL, 48 h: 9.84 ± 1.87 g/dL, 72 h: 9.17 ± 1.79 g/dL) with stable values at days 5 and 10. At admission, 63 patients (82%) had an Hb ≥ 10 g/dL, 10 (13%) had values of 8–10 g/dL, and 4 (5%) ≤ 8 g/dL. TnT elevation was observed after 24 h irrespective of admission Hb; however, patients with admission Hb values of 8–10 g/dL showed higher TnT levels at admission and at 24 h. No significant Hb-dependent pattern was identified. Stratification by ISS revealed no significant differences in Hb levels across time points.

**Correlation analysis of relevant biomarkers.** Baseline TnT strongly predicted subsequent TnT levels up to day 10. TnT correlated weakly positive with lactate during the first 72 h and weakly inverse with hemoglobin until day 5 (strongest at 24 h). Creatinine showed no association at admission but correlated moderately positively from 24–48 h through day 5, coinciding with a rise after 72 h. IL-6 peaked at 48 h and correlated weakly positive with TnT from admission to 72 h. NT-proBNP showed strong interactions with TnT from admission to day 5, while showing peak values around days 3–5. (Additional File 3 presents the 10-day kinetics of the respective biomarkers).

Subgroup Analysis – Early vs. delayed Troponin Elevation.

**Fig. 4:**
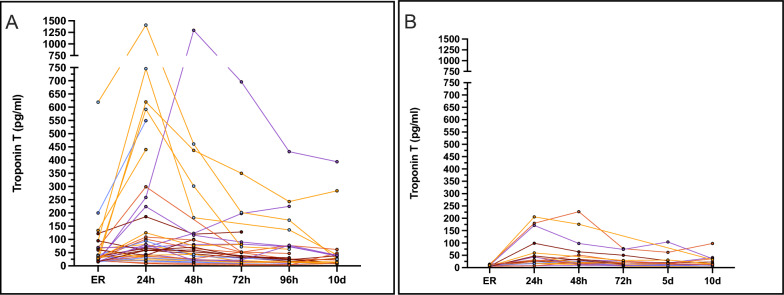
Individual Troponin Dynamics over 10 days in case of TnT Elevation at admission (**A**) and first elevation after 24 h or later (**B**) Serial measurements of TnT (pg/ml) are shown for each patient at admission to the emergency room (ER), and at 24 h, 48 h, 72 h, 96 h, and 10 days. Each line represents an individual patient course. TnT levels in both groups showed values over the 99th percentile cut-off at 14 pg/ml. For better visual comparability the axis configuration was equalized. In Group 2 (**B**), median TnT concentrations were 9.7 pg/ml at admission, 30.0 pg/ml at 24 h, 25.0 pg/ml at 48 h, 19.0 pg/ml at 72 h, 16.5 pg/ml at 5 days, and 17.0 pg/ml at 10 days

In the overall cohort, most patients (73%) exhibited elevated TnT values within 24 h. Several established risk factors were identified. To illustrate individual TnT patterns, an individual curve was generated for each patient (Fig. 4). Based on the timing of the first Tn elevation, patients were stratified into two groups: Group 1—elevation at admission (*n* = 34; 44%) and Group 2—elevation after 24 h (*n* = 20; 26%). Group 1 showed a marked rise within the first 24 h, followed by a gradual decline (Fig. 4a). In contrast, Group 2 exhibited a moderate but later increase, with peak values occurring at 24–48 h and subsequent decline over later intervals (Fig. 4b). Compared with Group 1, the Tn dynamics of Group 2 remained within a narrower range (0–250 pg/ml). Multiple comparisons using the Kruskal–Wallis test revealed significant differences in Group 1 between ER and 24 h (*p < 0.05) and in Group 2 between ER and 24–48 h (****p < 0.0001). In both groups, the highest values were observed at 24 h and 48 h after admission.

### Subgroup analysis of trauma dependent and independent risk factors for TnT elevation

Potential trauma-independent risk factors for Tn elevation subgroup analyses were repeated (subgroup distributions are found in additional file 4).

**Fig. 5:**
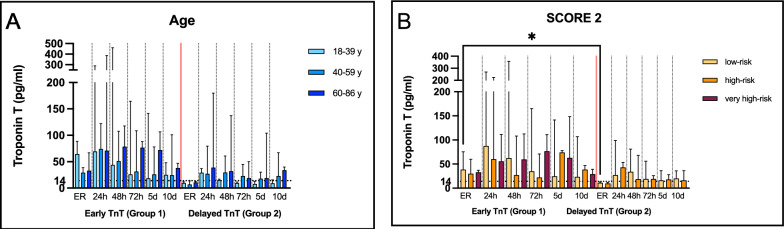
Subgroup analysis stratified by trauma-independent (**A**-**B**) risk factors.. ER = emergency room, h = hours, d = day. Data are presented as medians (bars) with interquartile ranges. Group 1 and 2 are divided by a read line. Since no normal distribution was detected, the Kruskal–Wallis test with Dunn’s correction for multiple comparisons was applied. Significance levels: *p < 0.05. (**A**) Age: No significant differences could be detected. (**B**) SCORE2: High-risk and very high-risk patients showed early elevation prolonged TnT elevation in Group 1. Group 2 had no very high-risk patients

**Fig. 6:**
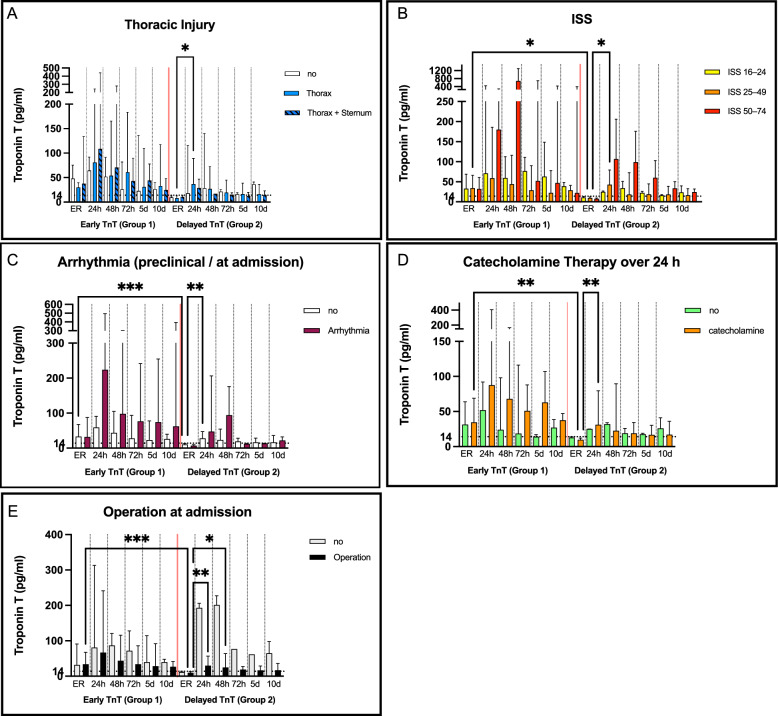
Subgroup analysis stratified by trauma-dependent (C–E) risk factors. ER = emergency room, h = hours, d = day. Data are presented as medians (bars) with interquartile ranges. Group 1 and 2 are divided by a read line. Since no normal distribution was detected, the Kruskal–Wallis test with Dunn’s correction for multiple comparisons was applied. Significance levels: *p < 0.05. (**A**) Thoracic Injury: Patients with thoracic injury exhibited higher TnT levels at ER and 24 h. Group 2 showed a significant dynamic within the first 24 h in case of thoracic trauma. (**B**) ISS: Higher ISS was associated with stronger TnT elevations, with the most pronounced values at 24–48 h. (**C**) Arrhythmia/Resuscitation: Patients with arrhythmia or resuscitation exhibited higher median values compared to other patients, without reaching significance. (**D**) Catecholamine Therapy over 24h:In Group 1, catecholamine therapy was associated with higher TnT levels at 24–72 h. In Group 2, catecholamine therapy after 24 h was associated with delayed TnT elevation. (**E**) Surgery at Admission: Surgical intervention was associated with higher TnT values in Group 2, with peak concentrations at 24–48 h

Age and SCORE2 (Fig. 5a-b): Older patients and those with a high SCORE 2 were more common in Group 1 and showed prolonged TnT elevation. A similar effect could be observed in patients ≥ 60 years in Group 2, while SCORE2 subgroups demonstrated no significant differences. Patients classified as very high risk were only present in Group 1. Thoracic Injury and ISS (Fig. 6a-b): In Group 1, thoracic trauma (especially sternal fracture) was associated with higher TnT levels. A delayed effect was still detectable in Group 2, in which 75% of patients had sustained thoracic trauma. A higher ISS correlated with greater TnT release in both groups, with a significant effect persisting in Group 2 for ISS ≥ 25. Arrhythmia (Fig. 6c): Arrhythmia at admission was associated with higher TnT levels compared to other risk factors, with levels peaking at 24–48 h in both groups. Nine of the 11 patients (81%) showed an increase already at admission and were therefore included in Group 1. Catecholamine Therapy (Fig. 6d): In Group 1, catecholamine therapy at admission was associated with higher TnT concentrations during the first 72 h. In Group 2, catecholamine use at admission did not influence delayed TnT release, but catecholamine therapy over 24 h was associated with elevated TnT levels. Surgery at Admission (Fig. 6e): Surgical intervention at admission was consistently associated with higher TnT levels in both groups, with peak concentrations observed at 24–48 h. The influence of surgery was significant in Group 2 of which 90% had surgery at admission; however, the non-operated group also demonstrated elevated TnT values due to arrhythmia at admission and severe thoracic trauma including cardiac contusion. Hemoglobin: Additional analyses were performed to evaluate the influence of blood loss. In both groups Hb at admission was in 74% ≥ 10 g/dL, Hb values ≤ 10 g/dL were observed in both groups, with severe anemia (Hb ≤ 8 g/dL) occurring in 15% of G2 compared with 2% of G1. After 24 h, Hb distributions were broadly comparable between groups: Hb ≥ 10 g/dL was present in 62% of G1 and 50% of G2, while Hb ≤ 8 g/dL persisted in 8% of G1 and 15% of G2. TnT elevation was more pronounced in the early group among patients with Hb ≤ 10 g/dL; however, this difference did not reach statistical significance. No clear temporal pattern of TnT changes was observed in Group 2.

**Troponin dynamics expressed as percentage change from the initial value:** To capture individual troponin dynamics, percentage changes from the initial value were calculated for each patient (additional file 5). Within the first 24 h, Group 2 showed substantially higher relative increases than Group 1 (median 258.5% vs. 113.5%). Group 2 also demonstrated greater changes across most risk-factor subgroups, with especially high median rises observed in patients with preclinical arrhythmia (1473%), an ISS ≥ 50 (1028%), Hb ≤ 8 g/dL at admission (749%), aged ≥ 60 (540%), ISS ≥ 25—49 (523.2%), thoracic trauma (430.9%), catecholamine therapy over 24 h (338.6%) and in case of an operation at admission (211.1%). In Group 1, the largest increase occurred in patients with ISS 50–74 (297%), preclinical arrhythmia (240.6%) and thoracic trauma (129.3%). Notably, these relative increases contrast with the absolute TnT values, where Group 2 remained within a narrow range (0–250 pg/ml), while Group 1 exhibited markedly higher early concentrations.

**Correlation analysis of relevant biomarkers:** Serial TnT values correlated strongly across time points in both groups (r = 0.7–0.9, *p* < 0.001). In Group 2, only NT-proBNP showed significant associations, with a correlation between NT-proBNP at admission and TnT after 24 h (r = 0.745, *p* < 0.05), as well as between NT-proBNP at 10d and TnT from 72 h to 10 d (r = 0.70–0.74, *p* ≤ 0.01). In Group 1, TnT correlated moderately with IL-6 and creatinine from 48 h to 5 d, moderately and inversely with hemoglobin from 24 to 72 h (r = –0.50 to –0.56, *p* < 0.01), and positively with lactate from 24 h to 5d (r = 0.50–0.59, *p* < 0.01).

### Subgroup Analysis of transthoracic echocardiography, ECG & complications

Shock and ARDS were recorded most frequently in the delayed elevation group (55% and 82%, respectively). New onset arrhythmias like atrial fibrillation occurred solely in patients with Tn elevation, with 9—10% in Group 1 and 2. In Group 1 additionally new AV and bundle branch block appeared in 15% and 18% respectively. One new bundle branch block was also detected in Group 2. Reanimation after admission was required in 15% of both TnT-positive groups. In early TnT elevation, pericardial (21%) and pleural effusions (29%) were frequent. Interestingly wall motion abnormalities, relaxation and diastolic dysfunction appeared across all groups. Group 1 and 2 were associated with higher mortality, especially in case of early TnT elevation.

Complications, TTE and ECG Abnormalities are comprehensively summarized in Table 1.

**Table 1:** Comparison of complications during hospital stay depending on TnT elevation

	Group 1—Early TnT Elevation (*n* = 34)	Group 2—Delayed TnT Elevation (*n* = 20)	Group 3—No TnT Elevation (*n* = 23)
Acute Kidney Failure	9 (26%)	6 (30%)	1 (4%)
Infection	18 (53%)	11 (55%)	3 (13%)
Shock	13 (38%)	11 (55%)	2 (9%)
ARDS	12 (35%)	9 (82%)	2 (9%)
	- 4 mild	- 7 mild	- 2 mild
	- 8 moderate	- 1 moderate	
		- 1 severe	
ECG Abnormalities	5 AV Block (15%)	1 bundle branch block (5%)	3 bundle branch block (13%)
	6 bundle branch block (18%)	6 repolarization abnormalities (30%)	5 repolarization abnormalities (22%)
	9 repolarization abnormalities (26%)		
New onset arrhythmias	3 atrial fibrillation/ flutter (9%)	2 atrial fibrillation (10%)	0
Later Reanimation	5 (15%)	3 (15%)	0 (0%)
TTE Abnormalities			
LVEF < 60%	3 (8%)	2 (10%)	1 (4%)
Pericardial effusion	7 (21%)	0	1 (4%)
Pleural effusion	10 (29%)	3 (15%)	4 (17%)
Wall movement abnormality	4 (12%)	2 (10%)	4 (17%)
Right ventricular dysfunction	3 (8%)	2 (10%)	3 (13%)
Relaxation Disorder	4 (12%)	2 (10%)	3 (13%)
Diastolic Disorder	4 (12%)	3 (15%)	3 (13%)
Mortality	7 (21%)	3 (15%)	0

Data were collected from electronic clinical records. Absolute numbers are presented, with percentages within each group shown in parentheses. Shock and ARDS were recorded when documented by the intensive care unit, which routinely applies the Shock Index or automated SAPS II/III scoring systems

## Discussion

This prospectively performed study visualized Tn patterns in polytrauma with regard to trauma-dependent and independent risk factors. Thoracic injury, preclinical arrhythmias or resuscitation were identified as significant influences on Tn increase, but also age ≥ 40 years, ISS ≥ 25, Hb ≤ 10 at admission and a high SCORE 2. Two distinct TnT kinetics were identified: an immediate elevation on admission with a broader range in Group 1, and a delayed elevation after 24 h with a more uniform course in Group 2. Interestingly, although Group 1 exhibited a wider overall range of values, the relative percentage increase per patient was markedly higher in Group 2. The “early” group was associated with the risk factors described above. In the “delayed” group, thoracic trauma, an ISS ≥ 25, and an Hb level ≤ 8 g/dL at admission showed a persistent effect; additionally, associations with surgery at admission and a prolonged need for catecholamines were observed.

Both groups showed increased risks of cardiac complications especially arrhythmias. These findings highlight the multifactorial cardiac pathophysiology in polytrauma and the need to refine diagnostic, monitoring, and treatment strategies, especially in trauma patients with a wide range or absence of cardiac symptoms. The aim of this study was to contribute to the ongoing discussion on the challenges of Tn interpretation and cardiac risk stratification in polytrauma, particularly in the era of high-sensitivity Tn assays. While the cardiology literature has widely debated the interpretation of high-sensitivity assays [10, 12, 13], the trauma literature rests on a dichotomous interpretation of Tn levels, because cardiac complications in trauma, unless life-threatening, are not a primary focus of acute management. Early Tn screening is mainly used to identify patients at risk of cardiac contusion following thoracic trauma [20], while neglecting the adverse risk factors for cardiac complications in polytrauma. In the past, Tn levels in trauma patients have been analyzed, demonstrating associations with greater injury severity and poorer outcomes [6, 13, 21, 22]. Furthermore, several studies have evaluated blunt chest trauma and cardiac injury [9, 24–26]; however, cardiac risk assessment remains largely unchanged and continues to rely on physician judgment. If cardiac contusion is ruled out due to normal Tn levels at admission, reevaluation is only conducted in rare cases. Although thoracic trauma occurred in 66% of patients and was significantly associated with Tn elevation and cardiac complications, in the present study we deliberately removed the thoracic trauma filter to study a broader patient population and to include the many co-factors present in polytrauma. Current literature on diagnostic criteria for cardiac contusion suggests that a combination of a normal ECG and normal Tn at admission effectively rules out cardiac injury [4]. The proposed diagnostic algorithm recommends repeating Tn measurement after 4–6 h in case of expected injury; if the result remains normal, further cardiac monitoring is not indicated [9]. Our study findings suggest a need to reconsider the time frame of current assessment strategies for cardiac contusion, as 75% of patients with delayed Tn elevation at 24 h experienced thoracic trauma. In some cases, additional Tn measurements were performed between admission and the 24-h mark—depending on clinical judgment—with variable results, indicating potential heterogeneity in early biomarker dynamics. This observed variability indicates that reliance on guideline-recommended early, threshold-based exclusion criteria may contribute to the underrecognition of myocardial injury.

Beyond direct trauma mechanisms, the concept of indirect myocardial injury in polytrauma has gained increasing attention. Cardiac dysfunction may result not only from direct injury but also from systemic inflammation, immune modulation, and therapy-associated stress [21, 27, 28]. Proinflammatory mediators (e.g., IL-6, tumor necrosis factor-α, interleukin-1β) contribute to endothelial dysfunction, myocardial apoptosis, and remodeling, highlighting a potential role for anti-inflammatory and oxidative stress–modulating therapies in myocardial protection [6, 7]. In our cohort, even patients with peripheral crush injuries demonstrated markedly elevated Tn levels and cardiac complications, supporting the presence of multiple pathophysiological mechanisms underlying Tn release in polytrauma, e.g. type 2 MI.

Moreover, patients with higher ISS showed a trend toward more pronounced Tn elevation. In the delayed group an ISS ≥ 25 showed a significant effect on Tn elevation. Generally, those with ISS ≥ 25 exhibited higher Tn concentrations, while patients with ISS ≥ 50 had higher median Tn values overall, with a wider range that often remained elevated up to day 10. This may be related to the higher frequency of operations, prolonged mechanical ventilation, blood loss, catecholamine exposure, and immune modulation, ultimately leading to increased cardiac stress and highlighting the need for further evaluation.

Next to inflammation, certain trauma mechanisms can also cause coronary artery dissection or transient occlusion due to vascular tearing, potentially resulting in type 1 or type 2 MI [29]. A previous case report has described atherosclerotic plaque rupture caused by blunt chest trauma, requiring coronary angiography and stent implantation [30], although the true incidence remains unclear. Differentiating ischemic from non-ischemic cardiac injury in polytrauma remains particularly challenging. In an aging population, attention to baseline cardiovascular risk factors is crucial. The polytrauma population, consisting predominantly of men with a mean age around 50 years, fits a high-risk profile for cardiovascular disease and MI. In our study, patients older than 40 years and those with a high SCORE2 tended to have higher Tn values at admission, although this did not reach statistical significance. Follow-up studies with bigger study populations are required. Nevertheless, this raises an important question: How can high-risk patients for coronary artery disease be accurately identified in the polytrauma setting, given the multiple potential causes of Tn elevation? Wereski et al. demonstrated that adjusting the interpretation thresholds for high-sensitivity Tn assays only marginally improved differentiation between type 1 and type 2 MI, and that acute myocardial injury often remains difficult to distinguish from type 1 MI [13]. In our study, several patients with Tn elevation also showed repolarization disorders, yet did not show clear signs of acute MI. Nevertheless, in some cases they required cardiologic interventions, including one case of coronary stent implantation and one case of pacemaker placement. More frequently, new-onset Episodes of atrial fibrillation and sinus tachycardia were also observed, resulting in new antiarrhythmic therapy in 6 cases and follow-up cardiologic co-management; however, the timing of these events varied considerably, which limited their predictability. This highlights the need for more specific risk stratification. Overall, the expanded cardiologic assessment applied in this study contributed to improved collaborative decision-making over time. Such integrative approaches should be encouraged and systematically incorporated into trauma care in the future.

Another new aspect in this study was the association of surgery at admission (60% of all patients) and a delayed Tn elevation (90% of Group 2). Previous studies have shown that non-cardiac surgery may be associated with perioperative myocardial infarction/injury (PMI), and cardiologists are currently discussing a new definition [17, 18, 20]. In a large prospective multicenter study, Puelacher et al. evaluated 7,754 patients aged ≥ 65 years or ≥ 45 years with cardiovascular disease undergoing non-cardiac surgery and found a 13.1% incidence of perioperative myocardial injury (PMI). Among these, 8.8% experienced major adverse cardiac events (MACE) and 10.5% died within one year. Outcomes varied markedly by the etiology of Tn elevation: patients after orthopedic, trauma, or spinal surgery most often exhibited type 2 MI, followed by extra-cardiac PMI (e.g., sepsis, pulmonary embolism) and type 1 MI, with corresponding one-year MACE rates of 17%, 30%, and 37%, and all-cause mortality rates of 17%, 35–45%, and 28%, respectively [20]. In the current study cohort, several overarching similarities with the findings of Puelacher et al. were observed. Cardiac causes of PMI included acute heart failure, evidenced by an early rise in NT-proBNP levels, and type 2 MI, suggested by an early association of blood loss with an inverse correlation with Hb in Group 1 and the high percentage increase in TnT in case of Hb ≤ 8 g/dL as well as requirement for catecholamine therapy (indicative of severe hypotension) in Group 2. Additionally, extra-cardiac mechanisms, particularly immune modulation, appear to contribute to PMI. This is supported by a pronounced increase in IL-6 within 24–48 h and the influence of an Injury ISS ≥ 25 in Group 2. These findings are especially noteworthy given the elevated one-year mortality associated with extra-cardiac PMI. Long-term survival data for our trauma patients are currently unavailable; however, this may be achievable in future studies. In the 2022 ESC guidelines, regular perioperative Tn monitoring is now recommended (Class I recommendation)—independently of trauma—to facilitate early identification of perioperative myocardial injury. Accordingly, patients with known cardiovascular disease (CVD), cardiovascular risk factors (including age ≥ 65 years), or symptoms or signs suggestive of CVD should have hs-Tn measurements performed before surgery, and at 24 and 48 h postoperatively, along with a 12-lead ECG prior to intermediate- or high-risk non-cardiac surgery [15]. These recommendations are consistent with the findings of this study and the identification of a cardiac risk group with delayed Tn elevation after 24 h. Additionally the TTE helped visualizing cardiac function.

This study has several limitations. The cohort mainly included predominantly male patients, which often had a higher basic risk, potentially biasing Tn elevation rates. Etiologic classification relied on clinical judgment rather than systematic invasive diagnostics e.g. coronary arteriography, risking misclassification. ECG evaluation could only be performed retrospectively. Overlapping conditions in severely injured patients further complicated interpretation. Echocardiography quality was occasionally limited by technical factors e.g. supine position, chest tube, postoperative free air following laparotomy. Another limitation of this study is the exclusive use of TnT; potential differences compared with the more cardiac-specific Tn I cannot be excluded and warrant further investigation. Finally, long-term cardiac outcomes and late Tn elevations warrant further study and validation in larger, external cohorts with advanced imaging. Follow-up studies with systematic ECG and echocardiographic analysis and a larger study population, as well as long-term re-evaluation, are planned for future research.

## Conclusion

This prospective study demonstrates that cardiac involvement in polytrauma is common and multifactorial. Overall, Tn elevation was more frequent in patients with thoracic trauma, preclinical arrhythmias or resuscitation and early blood loss with Hb ≤ 10 g/dl. Furthermore, patients aged ≥ 40 years and an ISS ≥ 25 tend to show higher values. Patients with an ISS ≥ 25, ≥ 60 years and those with high SCORE 2 demonstrated persistently elevated median TnT values over 10 days, while thoracic injuries—particularly with sternal fracture—were associated with early and shorter TnT elevation. Patients with immediate TnT elevation at admission showed greater value ranges, whereas those with delayed elevation after 24 h exhibited more uniform courses. In contrast, the relative percentage increase per patient was markedly higher in case of delayed TnT elevation after 24 h, showing a strong association with ISS ≥ 25, thoracic injury, surgical intervention, catecholamine use and an Hb ≤ 8 g/dL at admission. New onset arrhythmias and increased mortality appeared in both groups. Our findings support the 2022 ESC recommendation for routine perioperative Tn monitoring in case of polytrauma, identifying a cardiac risk subgroup with delayed Tn elevation after 24 h. TTE and continuous ECG proved valuable for detecting cardiac dysfunction and arrhythmias. These findings support incorporating troponin measurement into polytrauma management beyond early threshold-based exclusion of cardiac contusion to detect secondary cardiac involvement in the days following injury. This study underscores the urgent need for focused research on secondary cardiac damage in polytrauma patients, including systematic follow-up studies to evaluate potential long-term cardiac complications and to refine cardiac risk assessment strategies.

## Supplementary Information

Additional file1 (DOCX 17 KB)

Additional file2 (DOCX 16 KB)

Additional file3 (PDF 29 KB)

Additional file4 (DOCX 15 KB)

Additional file5 (DOCX 18 KB)

## Data Availability

No datasets were generated or analysed during the current study.
