# Supplementary material for: Beyond the initial impact: troponin patterns frequently reveal delayed cardiac injury in polytrauma patients
Source: World J Emerg Surg. 2026 Jan 31;21:10. doi: 10.1186/s13017-026-00672-4 (PMC12931084; doi:10.1186/s13017-026-00672-4)
Supplement: Supplementary file 1 — Additional file1 (DOCX 17 KB) [file 13017_2026_672_MOESM1_ESM.docx]

**Beyond the initial impact - Troponin patterns frequently reveal delayed cardiac injury in polytrauma patients**

**Additional File 1**

| Pre-existing conditions | Number of Patients (n=77) |
| --- | --- |
| Arrhythmias | 5 |
| Lung Artery Embolism | 1 |
| Coronary artery disease   - Past Acute Coronary Syndrome | 6  5 |
| Smoker | 10 |
| Alcohol dependence | 10 |
| Arterial hypertension | 27 |
| Diabetes Mellitus Type II | 5 |
| Hyperlipidemia | 8 |
| Cerebrovascular diseases | 6 |
| Peripheral arterial occlusive disease | 5 |
| Stroke | 7 |
| Chronic kidney disease | 2 |
| Chronic Obstructive Pulmonary Disease (COPD) | 4 |
| Heart insufficiency | 2 |
| Patients with known AP symptoms within the prior 4 weeks | 2 |
| Previous inflammation within the prior 4 weeks | 2 |
| Chronic inflammatory disease | 1 |
| Surgery or Intervention within the prior 4 weeks | 1 |
| Previous malignancy | 6 |
| Patients with available data on previous vascular/valve dysfunction | 5 |
| Recorded psychiatric diseases | 26 |

**Additional Table 1: pre-existing conditions**
