# Supplementary material for: Beyond the initial impact: troponin patterns frequently reveal delayed cardiac injury in polytrauma patients
Source: World J Emerg Surg. 2026 Jan 31;21:10. doi: 10.1186/s13017-026-00672-4 (PMC12931084; doi:10.1186/s13017-026-00672-4)
Supplement: Supplementary file 2 — Additional file2 (DOCX 16 KB) [file 13017_2026_672_MOESM2_ESM.docx]

**Beyond the initial impact: Troponin patterns frequently reveal delayed cardiac injury in polytrauma patients**

**Additional File 2**

| **Trauma Mechanism** | **Number of Patients (n=77)**  (% of total) | **Number of Patients with Troponin Elevation divided by timepoint**  (% within trauma mechanism group) |
| --- | --- | --- |
| **A fall from < 3m** | 11 (14 %) | 8 at admission (73%)  1 after 24h (9%) |
| **A fall from > 3m** | 21 (27%) | 10 at admission (48%)  7 after 24h (33%) |
| **Bike accident** | 2 (3%) | 1 after 24h (50%) |
| **E-Scooter accident** | 1 (1%) | 1 after 24h (100%) |
| **Stab wound** | 3 (4%) | 2 at admission (1 thoracic) (67%)  1 after 24h (33%) |
| **Motor bike injury** | 10 (13%) | 4 at admission (40%)  1 after 24h (10%) |
| **Pedestrian** | 7 (9%) | 4 at admission (57%)  3 after 24h (43%) |
| **Car crash** | 7 (9%) | 3 at admission (43%) |
| **Crush injury** | 9 (12%) | 1 at admission (11%)  4 after 24h (44%) |
| **Fist fight** | 2 (3%) | 0 |
| **Hit by train** | 3 (4%) | 2 at admission (67%)  1 after 24h (33%) |
| **Unclear** | 1 (1%) | 0 |

**Additional Table 2: Trauma Mechanisms**

The table presents the trauma mechanisms associated with troponin elevation in the two subgroups, G1 – TnT elevation at admission (n = 34) and G2 - TnT elevation after 24h (n = 20). For each subgroup, the number of patients and the corresponding percentage of the affected trauma mechanisms related to troponin elevation are reported.
