# Supplementary material for: Beyond the initial impact: troponin patterns frequently reveal delayed cardiac injury in polytrauma patients
Source: World J Emerg Surg. 2026 Jan 31;21:10. doi: 10.1186/s13017-026-00672-4 (PMC12931084; doi:10.1186/s13017-026-00672-4)
Supplement: Supplementary file 4 — Additional file4 (DOCX 15 KB) [file 13017_2026_672_MOESM4_ESM.docx]

**Beyond the initial impact: Troponin patterns frequently reveal delayed cardiac injury in polytrauma patients**

**Additional File 4**

| **Subgroup** | **n (Group 1)** | **n (Group 2)** |
| --- | --- | --- |
| **Age 18–39** | 8 | 5 |
| **Age 40–59** | 10 | 9 |
| **Age 60–86** | 16 | 6 |
| **SCORE2 low** | 14 | 11 |
| **SCORE2 high** | 11 | - |
| **SCORE2 very high** | 8 | - |
| **No thoracic trauma** | 10 | 5 |
| **Thoracic trauma** | 17 | 12 |
| **Thoracic + sternal trauma** | 7 | 3 |
| **ISS 16–24** | 11 | 5 |
| **ISS 25–49** | 19 | 13 |
| **ISS 50–74** | 4 | 2 |
| **No operation** | 13 | 2 |
| **Operation** | 21 | 18 |
| **No catecholamine ER** | 9 | 6 |
| **Catecholamine ER** | 25 | 14 |
| **No catecholamine 24h** | 10 | 3 |
| **Catecholamine 24h** | 24 | 17 |
| **No arrhythmia T0** | 25 | 18 |
| **Arrhythmia T0** | 9 | 2 |
| **SCORE2 moderate** | - | 9 |

**Subgroup distributions in Group 1 and Group 2**

Subgroup distributions for patients with early (Group 1, n = 34) and delayed (Group 2, n = 20) Troponin T (TnT) elevation. All subgroup analyses included the complete cohorts except for one patient in Group 1 without SCORE2 classification due to incomplete baseline data.
