# Supplementary material for: Beyond the initial impact: troponin patterns frequently reveal delayed cardiac injury in polytrauma patients
Source: World J Emerg Surg. 2026 Jan 31;21:10. doi: 10.1186/s13017-026-00672-4 (PMC12931084; doi:10.1186/s13017-026-00672-4)
Supplement: Supplementary file 5 — Additional file5 (DOCX 18 KB) [file 13017_2026_672_MOESM5_ESM.docx]

**Beyond the initial impact: Troponin patterns frequently reveal delayed cardiac injury in polytrauma patient**

**Additionial File 5**

| **Riskfactors for Troponin Increase by Subgroup** | **Time** | **Median percentage change from the initial value (%)** | **Range (%)** | **Mean**  **percentage change from the initial value (%)** | **Std. Dev. (%)** |
| --- | --- | --- | --- | --- | --- |
| **G2 preclinical arrhythmia** | **ER>24h** | 1473 | 1102 | 1473 | 779,2 |
| **G2 ISS 50–74** | **ER>24h** | 1028 | 1992 | 1028 | 1409 |
| **G2 age 60-86** | **ER>24h** | 540 | 1982 | 820,7 | 874,9 |
| **G2 25–49 ISS** | **ER>24h** | 523,2 | 1658 | 646,6 | 581,5 |
| **G2 thoracic trauma** | **ER>24h** | 430,9 | 1658 | 646,5 | 582,9 |
| **G2 age 40-59** | **ER>24h** | 338,6 | 1492 | 519,3 | 546,1 |
| **G2 Catecholamine over 24h** | **ER>24h** | 338,6 | 1982 | 646,8 | 643,3 |
| **G1 50–74 ISS** | **ER>24h** | 297 | 1433 | 549,7 | 654,3 |
| **G2** | **ER >24** | 258,5 | 1992 | 582,1 | 636,5 |
| **G1 preclinical arrhythmia** | **ER>24h** | 240,6 | 1407 | 556 | 567,2 |
| **G1 thoracic +sternal trauma** | **ER>24h** | 228,4 | 389,8 | 155,7 | 156 |
| **G2 operation at admssion** | **ER>24h** | 211,1 | 1992 | 559,9 | 627,4 |
| **G2 thoracic + sternal trauma** | **ER>24h** | 163,6 | 828,8 | 392,7 | 459,5 |
| **G1 catecholamine at admission** | **ER>24h** | 151,9 | 3053 | 407,9 | 696,2 |
| **G1 moderate-risk SCORE 2** | **ER>24h** | 144,4 | 848,6 | 231,2 | 281,5 |
| **G1 age 60-86** | **ER>24h** | 129,8 | 1491 | 277,2 | 393,3 |
| **G1 thoracic trauma** | **ER>24h** | 129,3 | 3039 | 480,4 | 808,4 |
| **G1 age 40-59** | **ER>24h** | 114,7 | 3053 | 422,1 | 915,3 |
| **G1** | **ER>24h** | 113,5 | 3057 | 307,9 | 605,3 |
| **G1 male** | **ER>24h** | 113,5 | 3057 | 311,5 | 667 |
| **G1 25–49 ISS** | **ER>24h** | 111,8 | 3054 | 303,9 | 695,2 |
| **G1 16–24 ISS** | **ER>24h** | 106,7 | 1514 | 227 | 424,2 |
| **G2 50–74 ISS** | **24h>48h** | 97,65 | 224,5 | 97,65 | 158,7 |
| **G1 operation at admission** | **ER>24h** | 86,1 | 3054 | 343,7 | 715,7 |
| **G1 high-risk SCORE 2** | **ER>24h** | 67,25 | 1455 | 260,9 | 496,7 |

**Additional Table 4: Troponin dynamics expressed as percentage change from the initial value, ordered by maximum observed increases.** The table displays risk factors associated with troponin increase in two subgroups, G1 (n = 34) and G2 (n = 20). For each subgroup and risk factor, the timespan of evaluation, the median percentage increase relative to the initial troponin value, the observed range, the mean percentage increase, and the standard deviation are reported.
