# Supplementary material for: Beyond the initial impact: troponin patterns frequently reveal delayed cardiac injury in polytrauma patients
Source: World J Emerg Surg. 2026 Jan 31;21:10. doi: 10.1186/s13017-026-00672-4 (PMC12931084; doi:10.1186/s13017-026-00672-4)
Supplement: Supplementary file 3 — Additional file3 (PDF 29 KB) [file 13017_2026_672_MOESM3_ESM.pdf]

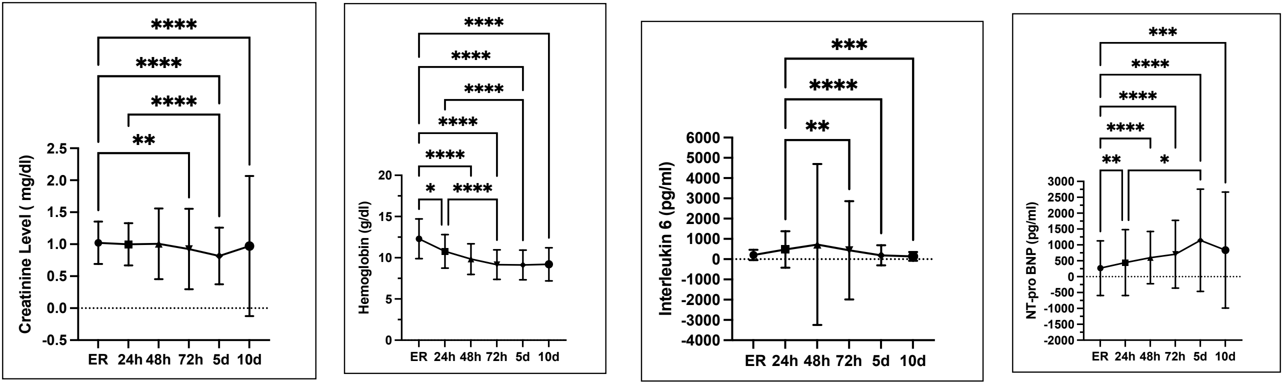

****
****
****
**
2.5
2.0
1.5
1.0
0.5
0.0
-0.5
Creatinine Level ( mg/dl)
ER
24h
48h
72h
5d
10d
****
****
****
****
****
*
****
20
15
10
5
0
Hemoglobin (g/dl)
ER
24h
48h
72h
5d
10d
***
****
**
6000
5000
4000
3000
2000
1000
0
-1000
-2000
-3000
-4000
Interleukin 6 (pg/ml)
ER
24h
48h
72h
5d
10d
***
****
****
****
**
*
3000
2500
2000
1500
1000
500
0
-500
-1000
-1500
-2000
NT-pro BNP (pg/ml)
ER
24h
48h
72h
5d
10d
